# Precise Control of Molecular Self‐Diffusion in Isoreticular and Multivariate Metal‐Organic Frameworks

**DOI:** 10.1002/cphc.201901043

**Published:** 2019-12-12

**Authors:** Thomas M. Osborn Popp, Ariel Z. Plantz, Omar M. Yaghi, Jeffrey A. Reimer

**Affiliations:** ^1^ Department of Chemistry, Kavli Energy NanoSciences Institute at Berkeley, and Berkeley Global Science Institute University of California-Berkeley Berkeley, California 94720 USA; ^2^ Department of Chemical and Biomolecular Engineering University of California-Berkeley Berkeley, California 94720 USA; ^3^ Materials Sciences Division, Lawrence Berkeley National Laboratory, Berkeley Berkeley CA 94720 USA

**Keywords:** diffusion, liquids, metal-organic frameworks, nuclear magnetic resonance, pulsed-field gradient

## Abstract

Understanding the factors that affect self‐diffusion in isoreticular and multivariate (MTV) MOFs is key to their application in drug delivery, separations, and heterogeneous catalysis. Here, we measure the apparent self‐diffusion of solvents saturated within the pores of large single crystals of MOF‐5, IRMOF‐3 (amino‐functionalized MOF‐5), and 17 MTV‐MOF‐5/IRMOF‐3 materials at various mole fractions. We find that the apparent self‐diffusion coefficient of *N*,*N‐*dimethylformamide (DMF) may be tuned linearly between the diffusion coefficients of MOF‐5 and IRMOF‐3 as a function of the linker mole fraction. We compare a series of solvents at saturation in MOF‐5 and IRMOF‐3 to elucidate the mechanism by which the linker amino groups tune molecular diffusion. The ratio of the self‐diffusion coefficients for solvents in MOF‐5 to those in IRMOF‐3 is similar across all solvents tested, regardless of solvent polarity. We conclude that average pore aperture, not solvent‐linker chemical interactions, is the primary factor responsible for the different diffusion dynamics upon introduction of an amino group to the linker.

## Graphical abstract

**Diffusion of guests** within the metal‐organic framework MOF‐5 is shown to linearly decrease as the isoreticular NH_2_‐functionalized linker is incorporated into the framework. This NH_2_ functionality is shown to slow the translational motion of guests by reducing the pore metrics of the framework, and not through hydrogen bonding.

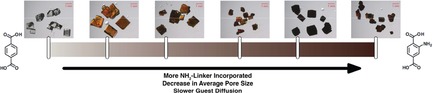

## 1 Introduction

Metal‐organic frameworks (MOFs) are porous, crystalline solids formed from joining organic linkers and metal‐oxide clusters.^1^ In many cases, careful choice of the organic linker and metal will result in the formation of a set of isoreticular MOFs which are different in molecular composition and pore metrics, but share identical structure‐types.^2^ An extension of the isoreticular principle is achieved by the mixture of two or more linkers or metals within one MOF of one topology to form what is known as a multivariate MOF (MTV‐MOF).^3^, ^4^ The isoreticular and multivariate concepts were first explored using MOF‐5,^5^ the archetypical MOF composed of tetrahedral Zn_4_O clusters connected by benzenedicarboxylate (BDC) units to form a cubic lattice. When 2‐aminobenzenedicarboxylate (NH_2_‐BDC) is used instead of BDC, the isoreticular derivative known as IRMOF‐3 is formed.^2^ The degree of mixing and the apportionment of linkers in MTV‐MOF‐5 materials have been documented, with solid state nuclear magnetic resonance (NMR) experiments in combination with simulations revealing that linkers mix on the length scale of nanometers.^6^

The porous and chemically tunable character of MOFs make them ideally suited for a number of practical applications. Optimization of MOFs for these applications often proceeds by isoreticular modification of a well‐studied base MOF structure, where a linker with a new functionality, yet identical connectivity, is used in place of the original linker, or mixed with the original linker at different stoichiometric ratios to form a multivariate material. This technique has been used in the development of MOFs for chemical separations, drug delivery, and heterogeneous catalysis.^7^, ^8^ An issue central to each of these applications is the impact of in‐pore molecular self‐diffusion on the material performance. Understanding what factors affect the self‐diffusion of molecules in MOFs is thus necessary for rational design of materials intended for these and other applications.

Previous work on the study of molecular self‐diffusion in MOFs has shown that the size of the molecule with respect to the pore metrics of the material has a significant impact on self‐diffusion.^8^, ^9^, ^10^ By mixing linkers in MTV‐MOFs, the average pore diameter and aperture size may be tuned between the discrete values attainable from single‐linker MOF materials. However, a MOF linker may be chosen that is capable of interacting with adsorbates by strong intermolecular forces such as hydrogen bonding, potentially complicating a rational intuition for predicting the relative self‐diffusion of molecules in MOFs based upon geometric arguments alone. It has been observed that hydrogen bonding plays a significant role in the adsorption of polar molecules in IRMOF‐3 at room temperature,^11^ suggesting that the capability to generate hydrogen bonds between linker and guest could be an influential factor in the self‐diffusion of polar solvents within MOFs. In this work, we use pulsed‐field gradient (PFG) NMR to measure the self‐diffusion of eight different solvents within MOF‐5, IRMOF‐3, and 17 different MTV‐MOF‐5/IRMOF‐3 materials at various linker stoichiometries. We elucidate the relative effects of intermolecular forces and pore metrics on self‐diffusion in these materials. Of the eight different solvents, *N*,*N‐*dimethylformamide (DMF) self‐diffusion is explored as a function of MOF composition.

## 2 Results and Discussion

Diffusion NMR experiments were performed at room temperature (295 K) using a homebuilt ^1^H PFG NMR probe, the configuration of which was based on an original design by Callaghan et al. and later reproduced by Wright and coworkers.^12^ Details of the probe and its construction are shown in the Supporting Information (Section 3).

**Figure 1:**
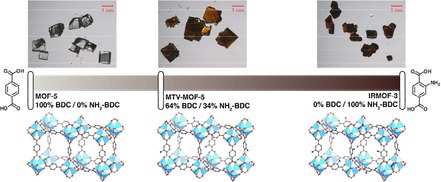
Molecular structure and corresponding optical microscope photographs of MOF‐5 (left), (MOF‐5)_0.64_(IRMOF‐3)_0.36_ (middle), and IRMOF‐3 (right).

Figure 1 depicts large single crystals (0.5–1.5 mm) of MOF‐5, IRMOF‐3 and MTV‐MOF‐5/IRMOF‐3, which were prepared according to literature conditions (see Supporting Information, Section 1).^3^, ^6^, ^13^, ^14^ All solvents used were anhydrous, and care was taken to minimize the materials’ exposure to atmospheric humidity, which has been demonstrated to degrade MOF‐5.^13^, ^15^ The BET areas as calculated from N_2_ isotherms of MOF‐5 and IRMOF‐3 are consistent with materials synthesized using optimal synthetic conditions (3488 m^2^ g^−1^ for MOF‐5, and 2520 m^2^ g^−1^ for IRMOF‐3, see Supporting Information, Figure 2).^13^, ^16^ The results of powder X‐ray diffraction on the crushed MOF crystals verify that all synthesized MTV‐MOF materials share their topology with MOF‐5 (Supporting Information, Section 2). To prepare the crystals for diffusion measurements, the MOFs were solvent‐exchanged with 5 mL of the target solvent five times. Excess extra‐crystalline solvent was removed prior to placement of crystals into a Kel‐F sample cell (Supporting Information, Section 3) in order to isolate the in‐pore solvent signal with PFG NMR.

**Figure 2:**
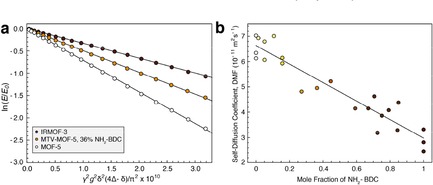
a) Diffusion attenuation curves for DMF within the pores of MOF‐5 (white), (MOF‐5)_0.64_(IRMOF‐3)_0.36_ (light brown), and IRMOF‐3 (dark brown). b) Self‐diffusion coefficient of DMF in 23 different batches of MTV‐MOF‐5 material shown with linear fit, ranging in linker mole fraction of NH_2_‐BDC from 0 to 100 %. MOF‐5 corresponds to 0 % NH_2_‐BDC mole fraction, while IRMOF‐3 corresponds to 100 %.

In‐pore solvent self‐diffusion coefficients were measured by fitting the exponential attenuation of signal as a function of increasing gradient strength using the PFG stimulated echo NMR pulse sequence. The modified Stejskal‐Tanner equation for the stimulated echo sequence with sine‐shaped gradient pulses is given by (1):

(1) $$E\left(g,\Delta \right)=exp\left(-D{\left(\frac{\gamma g\delta }{\pi }\right)}^{2}\left(4\Delta -\delta \right)\right)$$

where *D* is the self‐diffusion coefficient, *g* is the gradient strength, *γ* is the nuclear gyromagnetic ratio (*γ*(^1^H)=42.577 MHz T^−1^), *δ* is the gradient pulse width in time, and *Δ* is the diffusion interval.^17^ According to (1) a plot of log(*E*) vs. all terms other than *D* yields a linear curve with slope *D*.

Figure 2a details the signal attenuation curves for DMF in MOF‐5, IRMOF‐3, and a representative MTV‐MOF example, (MOF‐5)_0.64_(IRMOF‐3)_0.36_, at *Δ*=50 ms. Monoexponential diffusion is observed for DMF at all mole fractions of MTV‐MOF. Multiexponential diffusion might be expected if homogeneous linker domains in the binary MTV materials were much larger than the root mean squared displacement (RMSD) of a DMF molecule during the diffusion interval. The RMSD for molecules diffusing isotropically in three dimensions is a characteristic diffusive length scale given by (2):^18^

(2) $$\mathrm{RMSD}=\sqrt{6D\Delta }$$

As an example, for a solvent molecule diffusing with *D*=10^−11^ m^2^ s^−1^ for *Δ*=50 ms, the RMSD is 1.73 μm. The length scale of homogenous linker domains in mixed linker MTV‐MOF‐5 systems has been shown to be on the order of nanometers,^6^, ^19^ meaning that on the micron‐level diffusive length scales probed in this experiment, DMF is expected to experience an average pore environment weighted by the linker mole fraction. Accordingly, a linear trend is observed for the self‐diffusion coefficient of DMF as a function of linker mole fraction (Figure 2), where each point represents a measurement of DMF self‐diffusion in a separate batch of material. Significantly, when the self‐diffusion of DMF within a single batch of material was measured multiple times, it was found to be accurate to within ±5×10^−13^ m^2^ s^−1^ or less. However, when three separate batches of MOF‐5 and IRMOF‐3 material were synthesized and the self‐diffusion of DMF in each was measured, different values were measured for each batch. The average values for DMF in these two cases are *D*_*MOF‐5*_=6.53±0.47×10^−11^ m^2^ s^−1^ and *D*_*IRMOF‐3*_=2.86±0.44×10^−11^ m^2^ s^−1^, where the largest contribution to the error is batch‐to‐batch physical variability. The scatter in the diffusion coefficients of DMF among the unique binary‐linker MTV‐MOFs reflects the same variability as observed in the single‐linker MOF‐5 and IRMOF‐3 materials. It is possible that the presence of crystal defects at different concentrations between batches may be responsible for this variability, as the presence of defects has been proposed previously to be responsible for unexpected diffusion behavior in another MOF, Zn_2_(dopbdc).^20^

**Figure 3:**
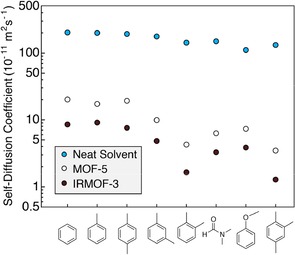
Self‐diffusion coefficients of various solvents neat (blue), within MOF‐5 (white), and within IRMOF‐3 (brown), arranged in order of increasing boiling point.

The linear relationship between the diffusion coefficient and the linker mole fraction in the MTV‐MOF materials demonstrates that the addition of the amino group to the linker has a significant effect on the translational motion of DMF within the pores. However, the mechanism by which this occurs may either be a molecular sieving effect caused by the steric bulk of the amino group, or a hydrogen bonding interaction between the amino group and DMF. The pore metrics between MOF‐5 and IRMOF‐3 are similar except for the pore aperture diameter, where the calculated aperture is 11.2 Å for MOF‐5 and 9.6 Å for IRMOF‐3.^2^ To determine whether the amino group affects diffusion primarily by pore aperture restriction or by hydrogen bonding, the diffusion coefficients of a series of pure polar and nonpolar solvents were measured in saturated in MOF‐5 and IRMOF‐3. All measurements were performed using the same batches of MOF‐5 or IRMOF‐3 for internal consistency. Figure 3 depicts the diffusion coefficients of several solvents in the three environments, arranged from lowest to highest solvent boiling point. The neat solvent diffusion coefficients are on the order of 10^−9^ m^2^ s^−1^, and tend to decrease with increasing boiling point. Inside the pores of MOF‐5, the self‐diffusion coefficients drop from their neat values by 1–2 orders of magnitude depending on the specific solvent. Among the nonpolar solvents, benzene, toluene, and para‐xylene show similar diffusion behavior in MOF‐5, but there is a significant difference between the xylene isomers, with para‐xylene diffusing the fastest, followed by meta‐xylene, then ortho‐xylene. This shape selectivity for translational motion in MOF‐5 has been attributed to steric interactions between the linkers and the xylene methyl groups, causing each isomer to have a different preferential alignment within the pores and different self‐diffusion coefficients.^21^ 1,2,4‐trimethylbenzene displays a self‐diffusion coefficient similar to ortho‐xylene, likely due to the presence of its 1‐ and 2‐methyl substituents yielding a preferential ordering effect similar to that of ortho‐xylene. Anisole displays a drop in diffusion coefficient in MOF‐5 of about an order of magnitude compared to benzene, toluene, and para‐xylene. Despite DMF having the lowest molar mass of all the tested solvents, it shows a large drop in the self‐diffusion coefficient in both MOF‐5 and IRMOF‐3. It has been suggested that DMF coordinates transiently to Zn^2+^ within the metal clusters of MOF‐5, meaning it would have a lower diffusion coefficient within MOF‐5 than a non‐coordinating molecule.^22^ While each solvent may have a different reason for its change in self‐diffusion between the neat and MOF‐5 environments, the addition of the amino group to the linker acts as a near constant change in self‐diffusion across all solvents tested – the average reduction in self‐diffusion between MOF‐5 and IRMOF‐3 is a factor of 2.24±0.34, regardless of solvent polarity. DMF and anisole are the only solvents amongst this selection that can participate in hydrogen bonding with the amino group on the linker, yet they experience similar a decrease in their self‐diffusion coefficient compared to the nonpolar solvents. The constant factor of 2.24±0.34 is likely a consequence of the similar size of the molecules tested, as significantly larger molecules should experience an even greater reduction in diffusion coefficient between the pores of MOF‐5 and IRMOF‐3. We conclude that the addition of linker‐based amino groups does not affect the self‐diffusion of solvents via hydrogen bonding at room temperature. Rather, it is the reduction in pore aperture size that leads to a steric interaction affecting both polar and nonpolar solvents similarly.

## 3 Conclusions

These results suggest that the primary mechanism that controls the self‐diffusion of molecules within MOFs is the pore geometry. Surprisingly, intermolecular forces such as hydrogen bonding between the linker and diffusing molecule appear to have a significantly lesser role on in‐pore self‐diffusion. As pore metrics are highly tunable in MOFs via isoreticular and multivariate methods, the translational motion of molecules can be tuned precisely by pore shape and geometry. Small isoreticular changes, such as the addition of an –NH_2_ group to a linker, can exert a large effect on self‐diffusion uniformly across both polar and nonpolar solvent molecules, and this effect can be precisely modulated by mixing linkers at various mole fractions. These results suggest that the precise control of molecular diffusion in MOFs can, to a first approximation, be governed by the pore geometries that derive from linker shape. This should make the purposeful design of MOFs for applications such as small molecule separations, catalysis, and drug delivery, more straightforward.

## Supplementary Information

Details on the preparation and characterization of samples, construction of the NMR hardware, execution of NMR experiments, and data analysis may be found in the supplementary information.

## Supporting information

As a service to our authors and readers, this journal provides supporting information supplied by the authors. Such materials are peer reviewed and may be re‐organized for online delivery, but are not copy‐edited or typeset. Technical support issues arising from supporting information (other than missing files) should be addressed to the authors.

Supplementary
