# Supplementary material for: Precise Control of Molecular Self‐Diffusion in Isoreticular and Multivariate Metal‐Organic Frameworks
Source: Chemphyschem. 2019 Dec 12;21(1):32–5. doi: 10.1002/cphc.201901043 (PMC7004185; doi:10.1002/cphc.201901043)
Supplement: Supplementary file 1 — Supplementary [file CPHC-21-32-s001.pdf]

CHEMPHYSCHEM

Supporting Information

© Copyright Wiley-VCH Verlag GmbH & Co. KGaA, 69451 Weinheim, 2019

Precise Control of Molecular Self-Diffusion in Isoreticular and Multivariate Metal-Organic Frameworks

Thomas M. Osborn Popp, Ariel Z. Plantz, Omar M. Yaghi, and Jeffrey A. Reimer*

Supplementary Information

Precise control of molecular self-diffusion in isoreticular and multivariate metal-organic frameworks

Thomas M. Osborn Popp,[1,2,3] Ariel Z. Plantz,[2] Omar M. Yaghi,[1,3] Jeffrey A. Reimer[2,3,*]

[1]Department of Chemistry, Kavli Energy NanoSciences Institute at Berkeley, and Berkeley Global Science Institute, University of California-Berkeley, Berkeley, California 94720

[2]Department of Chemical and Biomolecular Engineering, University of California, Berkeley, California 94720, USA University of California-Berkeley, Berkeley, California 94720

[3]Materials Sciences Division, Lawrence Berkeley National Laboratory, Berkeley, CA 94720

*Correspondence to: reimer@berkeley.edu

Table of Contents

Section 1: Syntheses of Materials……………………………………………………………..……………...2

Section 2: Material Characterization…………………………………………………………..……………...3

Section 3: PFG NMR Probe Design, Calibration, and Implementation…………………………………..…..9

Section 4: PFG NMR Signal Attenuation Plots and Tables of Self-Diffusion Coefficients..…..…………….15

Section 5: References…………………………………………………………………………..…………….23

Section 1: Syntheses of Materials

Chemicals used in this work. Zinc nitrate hexahydrate ($Zn(NO_3)_2{\cdot}6H_2O$), terephthalic acid (benzene-1,4-dicarboxylic acid or BDC), 2-aminoterephthalic acid (NH_2-BDC), anhydrous *N,N*-diethylformamide (DEF), dimethylformamide (DMF), dichloromethane (DCM), DCl, DMSO-d_6, toluene, p-xylene, o-xylene, m-xylene, anisole, 1,2,4-trimethylbenzene, were obtained at highest purity from Sigma-Aldrich and used without further purification.

MOF-5 Synthesis. Zinc nitrate hexahydrate (3.5 g, 18.5 mmol), BDC (656 mg, 3.95 mmol), and DEF (100 mL) were combined in a 200 mL glass container with a teflon-lined cap and sonicated until the BDC was fully dissolved. The resulting solution was heated at 85˚C for 72 hours or until clear, cubic crystals had formed. The crystals were then solvent exchanged with DMF every 12 hours for three exchanges, and the headspace of the vials were backfilled with dry N_2 after each exchange.

IRMOF-3 Synthesis. Zinc nitrate hexahydrate (3.5 g, 18.5 mmol), NH_2-BDC (715.5 mg, 3.95 mmol), and DEF (100 mL) were combined in a 200 mL glass container with a teflon-lined cap and sonicated until the NH_2-BDC was fully dissolved. The resulting solution was heated at 95˚C for 72 hours or until brown, cubic crystals had formed. The crystals were then solvent exchanged with DMF every 12 hours for three exchanges, and the headspace of the vials were backfilled with dry N_2 after each exchange.

MTV-MOF Synthesis. Zinc nitrate hexahydrate (.7 g, 3.7 mmol), DEF (20 mL), and the desired ratio of BDC:NH_2-BDC (.79 mmol total) were combined in a 20 mL scintillation vial with a teflon-lined cap and sonicated until all constituents were fully dissolved. The resulting solution was heated at 85˚C-95˚C for 72 hours or until cubic crystals had formed. The crystals were then solvent exchanged with DMF every 12 hours for three exchanges, and the headspace of the vials were backfilled with dry N_2 after each exchange.

Sample Preparation for Diffusion Measurements. Crystals of a single batch of MOF-5 and a single batch of IRMOF-3 were solvent exchanged from DMF into other test solvents. These crystals were exchanged into their target solvent (toluene, p-xylene, o-xylene, m-xylene, anisole, or 1,2,4-trimethylbenzene) through three consecutive exchanges every 12 hours using 5 mL of solvent each time. Once the samples were prepared in their respective solvents, the crystals were dried gently with a kimwipe to remove excess solvent. The crystals were then into homebuilt Kel-F sample cells for measurement.

Section 2: Material Characterization

Analytical Instrumentation. Powder x-ray diffraction patterns (PXRD) were recorded using a Bruker D8 Discover GADDS Powder X-ray diffractometer (Cu K_α radiation λ= 1.54056 Å). Solution ^{1}H NMR spectra were acquired on a Bruker AVB-400 NMR spectrometer. N_2 sorption isotherms of MOF-5 were measured on a Quantachrome Quadrasorb instrument, held at 77 K using a liquid nitrogen bath. Ultra-high purity grade N_2 was used for the adsorption experiments. Solid state UV-Vis spectra were acquired using a Fischer Scientific EVO 300 spectrometer.

Material Digestion and ^{1}H Solution State NMR. Digestion NMR was performed to determine final linker ratios and crystal structure respectively (Figure 3.1). A few crystals were taken from each MTV-MOF batch and patted dry with a kimwipe, then digested using dilute DCl solution (200 µL of 20% DCl/D_2O solution and 10 mL DMSO-d6). After dissolution, the solution was examined directly by ^{1}H NMR. ^{1}H NMR of the digested MTV-MOF-5 crystals (400 MHz, DMSO-d6): BDC δ: 8.00 (s, 4H). NH_2-BDC δ: 7.03 (d, 1H), 7.38 (s, 1H), 7.74 (d, 1H). Relative ratios of the two linkers for all synthesized MTV-MOF materials are given in Supplementary Figure 1.

Activation of Samples for N_2 Sorption Measurements. Samples of MOF-5 and IRMOF-3 were made in sufficient quantity that N_2 sorption measurements could be performed to assess the quality of our material preparation and handling techniques across all samples. Crystals were solvent exchanged from DMF to DCM three times. MOF-5 was activated under vacuum at room temperature for 72 hours, while IRMOF-3 was activated under vacuum at 75˚C for 72 hours. N_2 sorption data is depicted in Supplementary Figure 2. Points for the calculation of the BET area were chosen between

Sample Preparation for PXRD. To prepare samples for powder X-ray diffraction analysis, a few large single crystals of MOF that had been solvent-exchanged to DMF were removed from solvent and crushed into a powder using a mortar and pestle. Supplementary Figure 3 shows the PXRD patterns for different batches of MOF, labeled with the percent composition of NH_2-BDC linkers. Different batches with the same BDC/NH_2-BDC ratios are distinguished with an A or B label. Variations in the diffraction peak intensity between samples occur due to preferential crystal orientations resulting from incomplete crushing of the crystals. However, all peak positions are consistent with the simulated patterns for MOF-5 and IRMOF-3, confirming the isostructural nature of the synthesized MTV-MOFs.

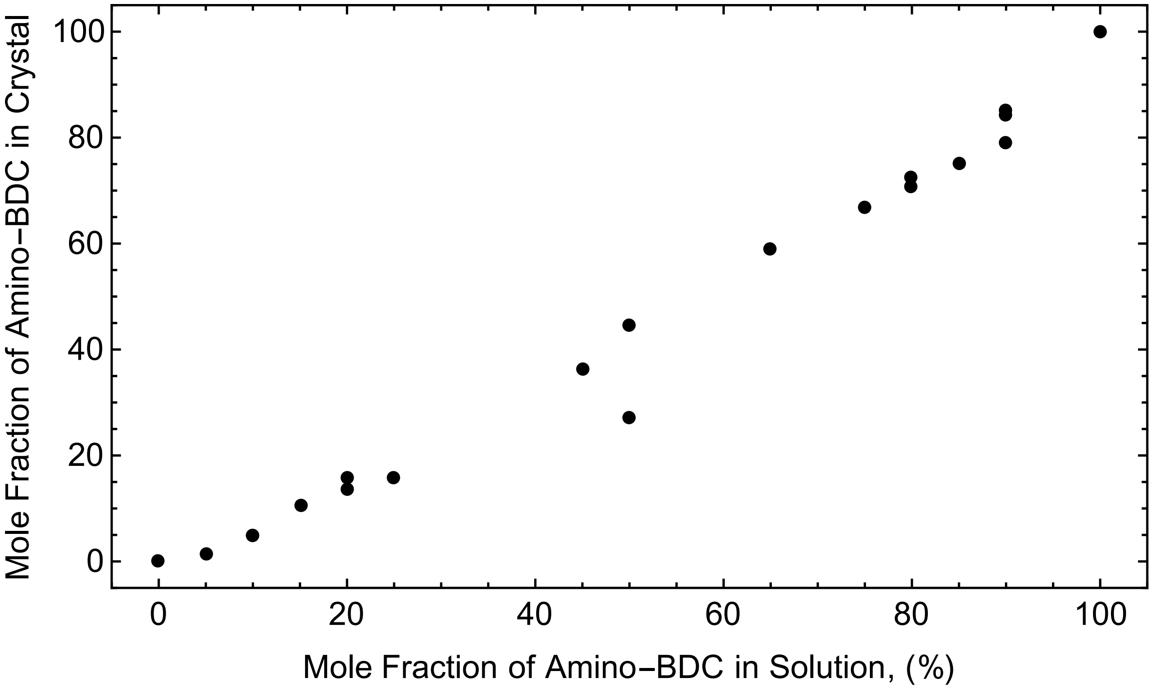

Supplementary Figure 1. Comparison of the molar ratio of linkers (BDC and NH_2-BDC) used in the MTV-MOF synthesis solution to the observed linker ratio incorporated into the crystal, as measured by digestion of the material and subsequent ^1H solution NMR. In a few cases, significant differences between the mole fraction of NH_2-BDC incorporated into the crystals versus the ratio of linkers in the synthesis solution were observed, highlighting the importance of characterization by acid digestion and ^1H NMR.

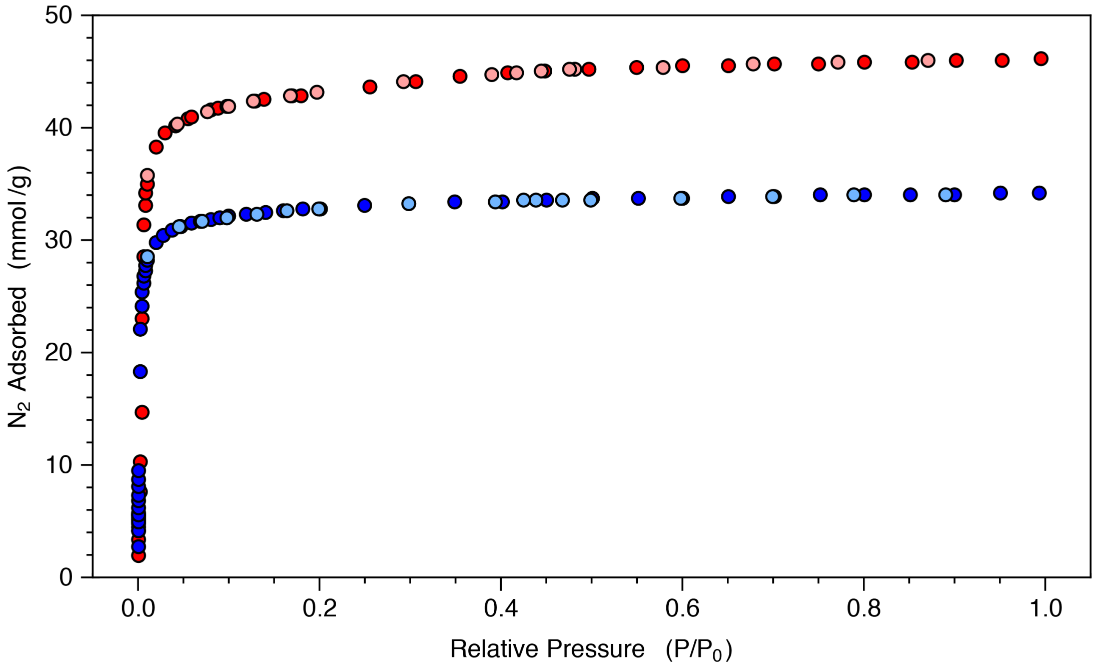

Supplementary Figure 2. N_2 adsorption isotherms of MOF-5 (red circles: adsorption, light red circles: desorption) and IRMOF-3 (blue circles, adsorption, light blue circles: desorption) at 77K. The calculated BET area was found to be 3488 m^2g^{-1} for MOF-5, and 2520 m^2g^{-1} for IRMOF-3.

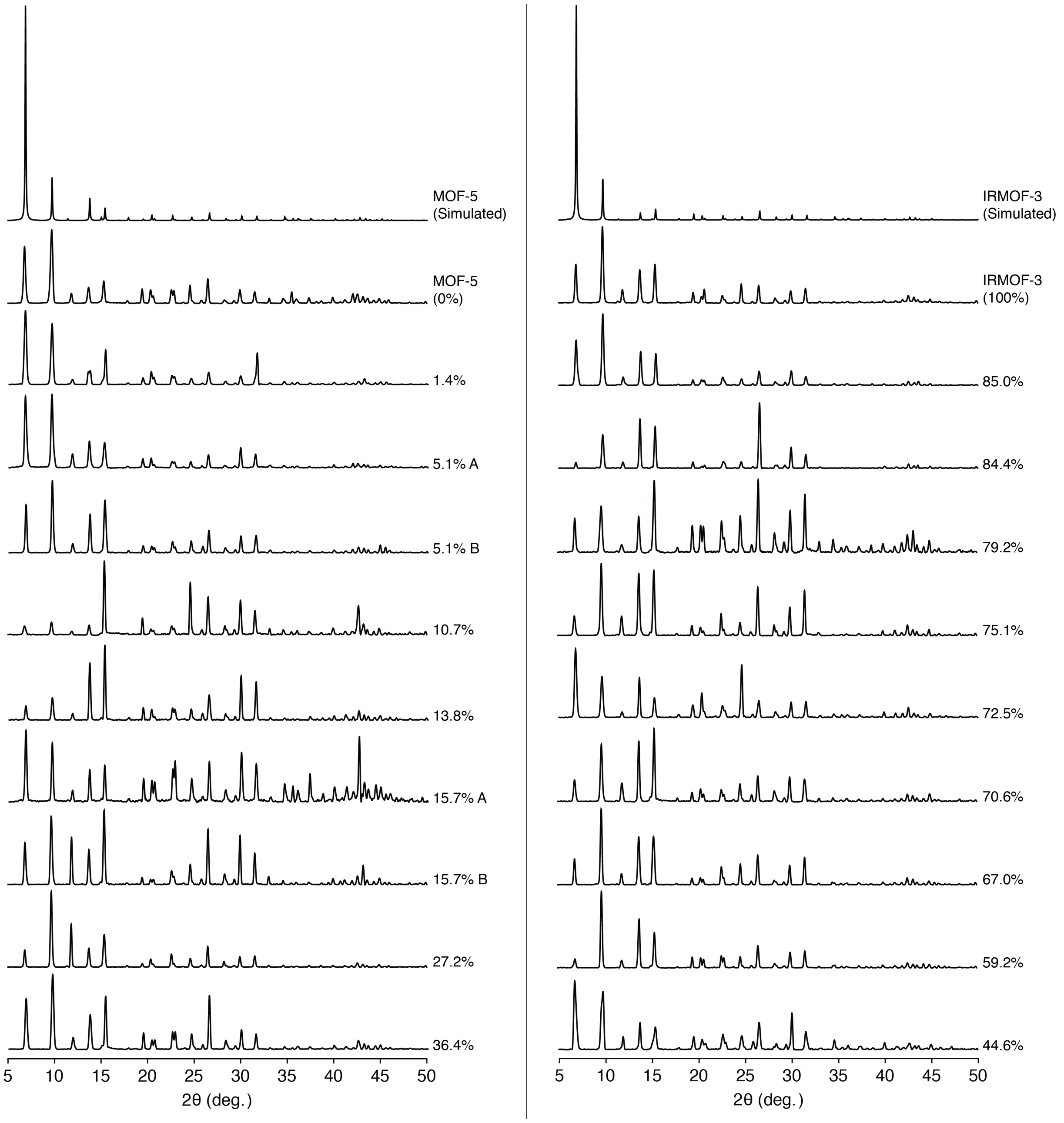

Supplementary Figure 3. PXRD patterns of crushed MOF-5, IRMOF-3, and MTV-MOF crystals (labeled by % composition of NH_2-BDC), compared to the simulated patterns for MOF-5 and IRMOF-3.

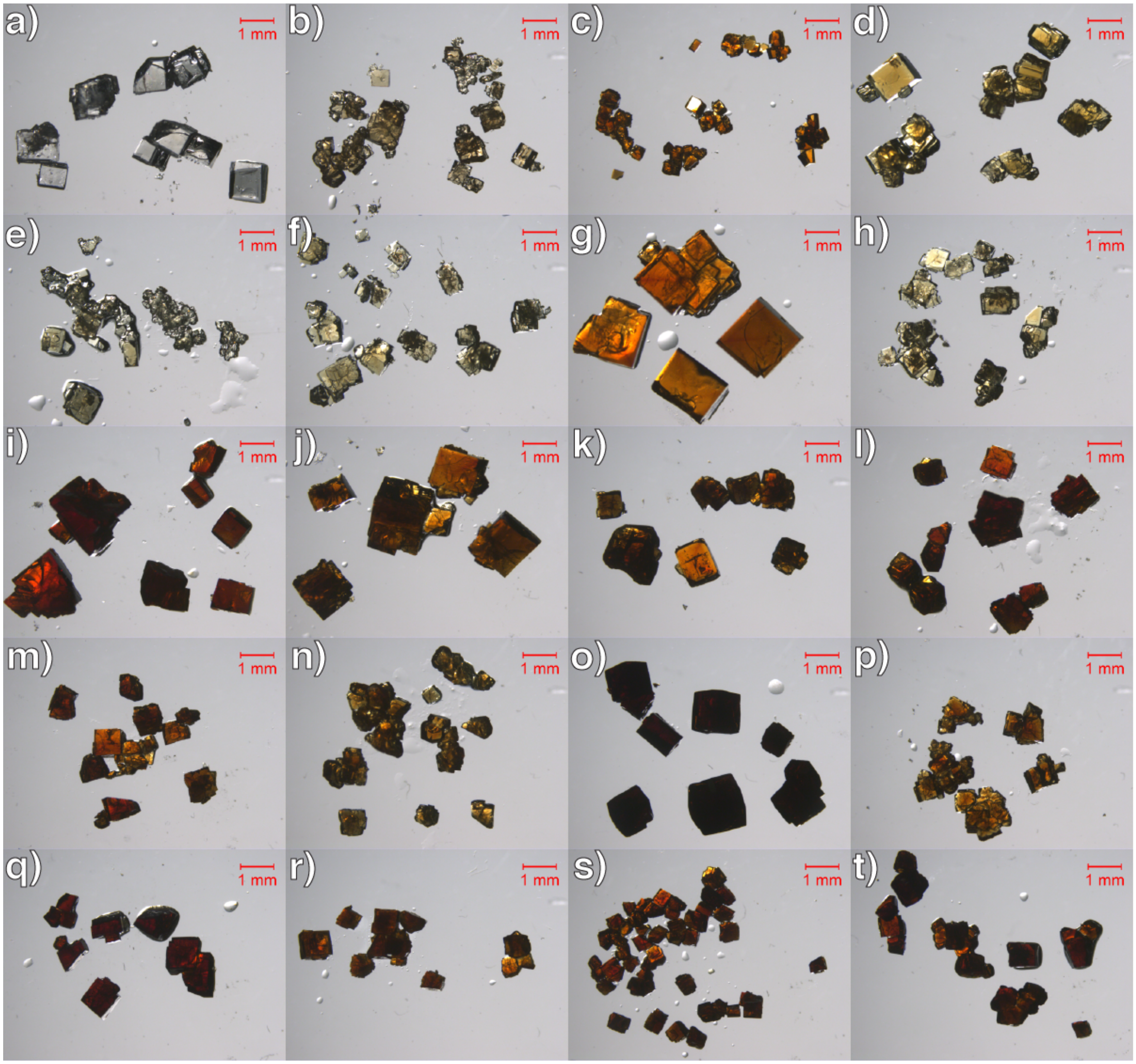

Supplementary Figure 4. Optical microscope images of MTV-MOF crystals ordered by increasing percent of amino-bdc linker: a) 0% b) 1.4% c) 5.1% d) 5.1% e) 10.7% f) 13.8% g) 15.7% h) 15.7% i) 27.2% j) 36.4% k) 44.6% l) 59.2% m) 67% n) 70.6% o) 72.5% p) 75.1% q) 84.4% r) 85% s) 95.1% t) 100%.

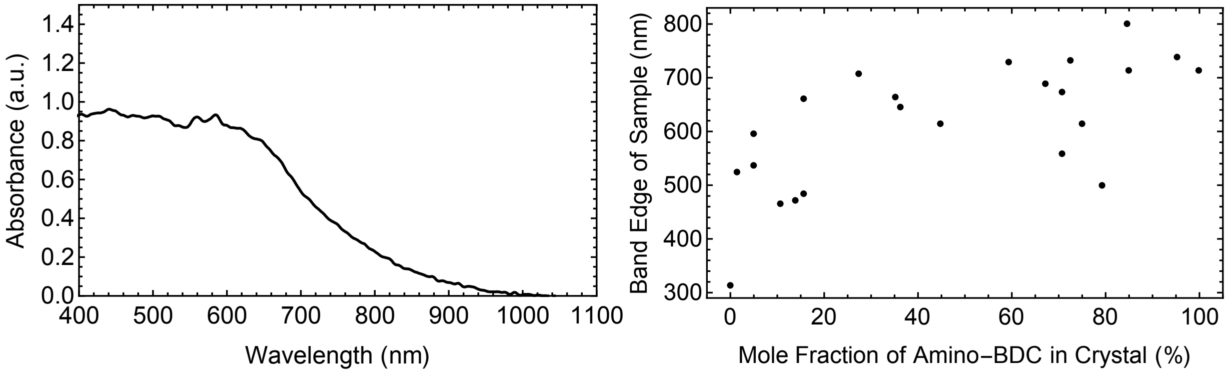

Supplementary Figure 5. On left, solid state UV-Vis spectrum of IRMOF-3, shown as a characteristic example of the absorbance profiles seen across all samples tested. Samples differed by maximum absorbance and band edge, but all had a similar shape. Pictured on right is a plot of the band edge of MTV-MOFs as a function of linker mole fraction. The band edge value was obtained by measuring the wavelength at which the absorbance was half of the maximum.

Section 3: PFG NMR Probe Design, Calibration, and Implementation

Probe Design and Construction. The probe body of a Bruker z-33f HP 300 MHz (89 mm diameter) solids probe was used to house the probe hardware. Parts for the probe and probehead assembly were modeled in Solidworks. The probehead consists of a Delrin base onto which is mounted the coil support assembly. Using 26 AWG bare copper wire, a 3-turn RF solenoid was wrapped around a machined Macor tube part with a 3 mm inner diameter and a threaded coil slot. The RF coil was centered within a SLA 3D-printed gradient coil support onto which was wound 25 turns of enameled 27 AWG copper magnet wire. During winding, the wires were painted with Devcon 2-Ton epoxy to adhere them strongly to the support. The gradient coil was then epoxied to its housing within a 3D-printed carbon-nylon composite support which was then secured to the Delrin probehead base by threaded nylon screws. The 3D-printed support was designed with space for airflow around the gradient coil for cooling. The RF solenoid coil leads were soldered to strands of PTFE-insulated 22 AWG wire, which connected them to 2 Johanson 0.5-10 pF variable capacitors. The enameled gradient coil leads were twisted and then epoxied to the probehead, and soldered to twisted strands of 10 AWG insulated copper wire, which was epoxied to the probe body to prevent mechanical vibrations. The coil assembly was covered with a Teflon cowling that tightly couples to the shield cover via a Viton O-ring. The 90° pulse time of the RF coil at 299.8 MHz at 20 W is 3.6 μs. The gradient coil has an inductance of 22 μH and a resistance of 1.9 Ω. A sample inserter with exchangeable Kel-F sample cells (3 mm O.D.) was made for quick and efficient sample changes.

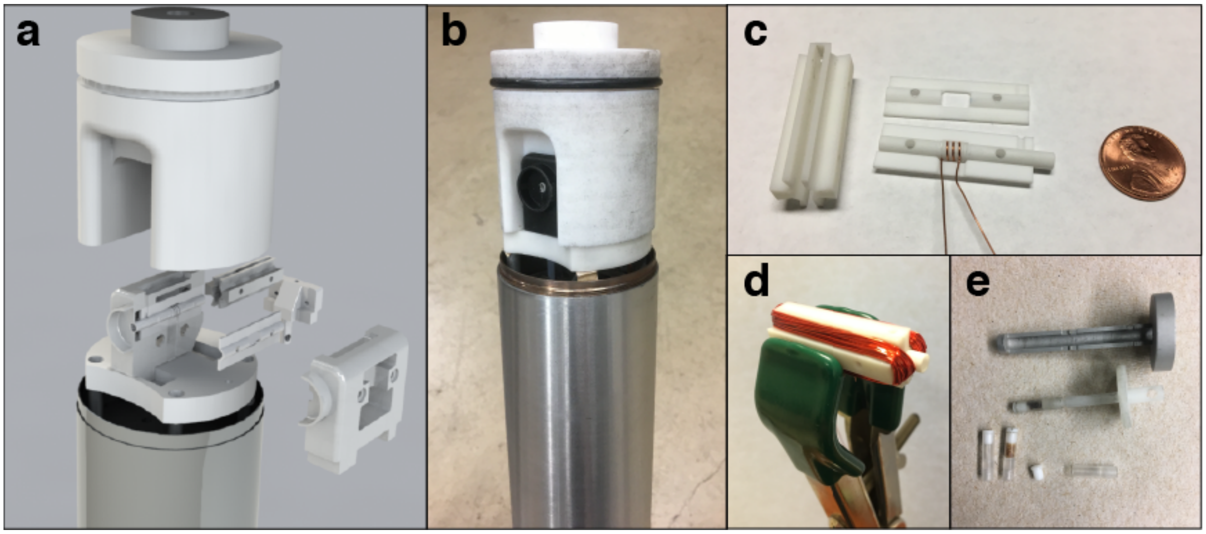

Supplementary Figure 6. a) Design of the ^{1}H PFG NMR probe, shown in exploded view. b) Assembled probe. c) Gradient coil supports with RF coil and sample tube before assembly, with U.S. penny coin for scale. d) Assembled gradient coil and RF coil core. e) Aluminum sample depth gauge with nylon sample inserter and exchangeable Kel-F sample cells.

Supplementary Figure 6 shows the schematic design and assembly of the probe. The gradient coil in this probe configuration is a quadrupolar array of four wire bundles surrounding a solenoid RF coil (Figure 1c,d). This design provides the ability to produce strong magnetic field gradients at relatively low current values using a small gradient coil, which reduces eddy current effects. Probes built using this design have been able to achieve gradient strengths of up to 50 T/m using only air-cooling.[1,2] However, even when achievable, the practical application of these high-magnitude gradient pulses to PFG NMR can be difficult, requiring an extremely rigid probe assembly to prevent mechanical vibrations induced by the Lorentz force during pulsing, as well as very precise matching of the gradient pulses used in the PFG NMR experiment.[3] To avoid these issues, the homebuilt probe implemented in this study was used at gradient strengths up to only 5 T/m. The probe was verified to reproducibly measure self-diffusion coefficients with high precision.

Calibration of the Probe for PFG NMR. NMR experiments were performed using an Oxford Instruments wide-bore 7.05 T superconducting magnet, a Tecmag Scout with a single channel gradient cluster, a Herley-AMT 3303 high power RF amplifier, and an AETechron 2105 gradient amplifier. The gradient amplifier and gradient coil were impedance-matched by selecting resistance and capacitance values for the compensation circuit within the AETechron amplifier that resulted in minimal ringing at the edges of a square gradient pulse.

The operating conditions for the probe were found by measuring the diffusive signal attenuation of a solution of 20,000,000 MW polystyrene in CCl_4 (80 mg/mL).[1,3] To quote a maxim stated by Callaghan,[1] “All artifacts cause excess echo attenuation, never reduced echo attenuation.” Due to the extremely slow self-diffusion (10^{-15} – 10^{-16} m^2s^{-1}) of the polymer, minimal attenuation should be observed even at very high gradient pulse amplitudes, but attenuation due to mechanical vibration or other issues will manifest clearly. The best operating conditions for the probe were found when using sine-shaped gradient pulses with the stimulated echo diffusion pulse sequence (PFG-STE), showing no artefactual attenuation below 5 T/m, indicating that mechanical vibrations occur at higher gradient amplitudes that interfere with accurate data collection.

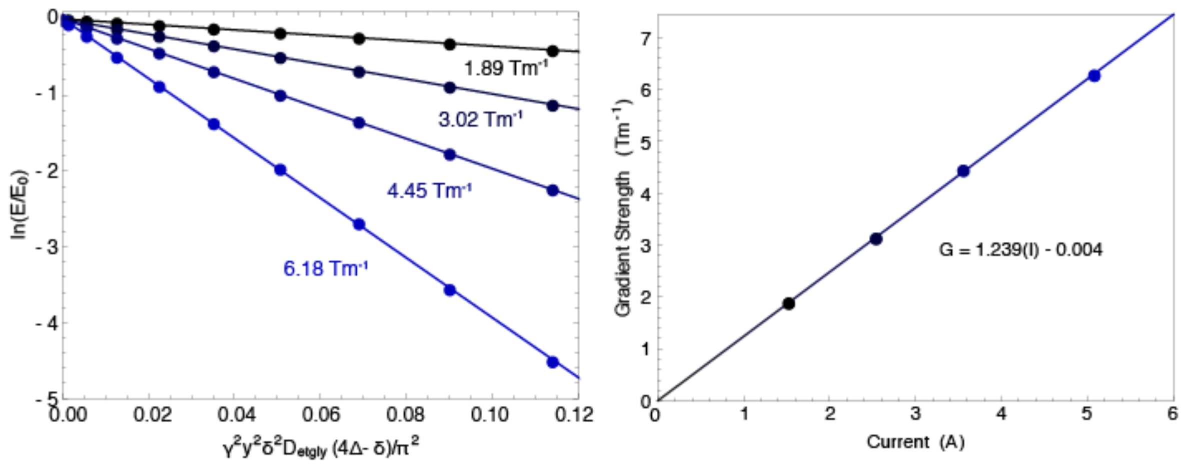

Supplementary Figure 7. Diffusive attenuation of ethylene glycol at 23˚C, as a function of increasing current applied to the gradient coil.

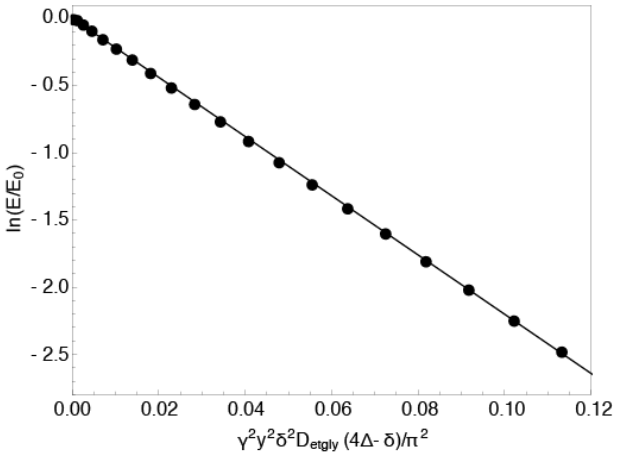

Supplementary Figure 8. Diffusive attenuation of ethylene glycol at 23˚C using 3.8 A of current, yielding a maximum gradient strength of 4.7 T.

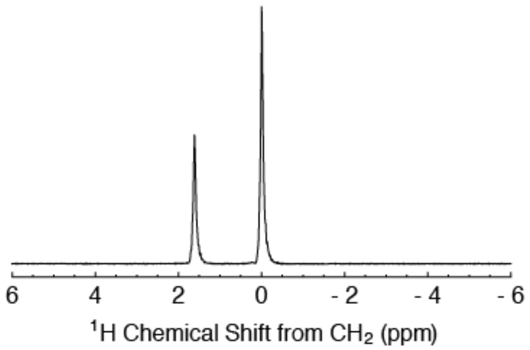

Supplementary Figure 9. ^{1}H spectrum of dry ethylene glycol. The field profile across the sample is inherently difficult to shim due to the sample shape and the configuration of the probe.

The gradient strength was calibrated by measuring the PFG-STE signal attenuation curve for ethylene glycol that was dried over 3 Å molecular sieves (Supplementary Figure 7). Ethylene glycol has a temperature-dependent separation between its CH_2 and OH resonances, making it useful for temperature calibration.[4,5] Additionally, the self-diffusion of ethylene glycol at a range of temperatures near room temperature has been well documented.[6] A major source of error in any diffusion measurement is the sample temperature, making ethylene glycol an excellent standard to calibrate the strength of gradients due to the ability to check the temperature *in situ*. The modified Stejskal-Tanner equation for the PFG-STE sequence with sine-shaped gradient pulses is given by (1):

$$\ln(E(g,\Delta)) = -D\left(\frac{\gamma g \delta}{\pi}\right)^2 (4\Delta - \delta) \quad (1)$$

where *D* is the self-diffusion coefficient, *g* is the gradient strength, *γ* is the nuclear gyromagnetic ratio ($\gamma(^1H) = 42.577$ MHz T^{-1}), *δ* is the gradient pulse width in time, and *Δ* is the diffusion interval .[7,8] In order to solve for the maximum gradient strength used in the experiment, *g* is set to *yG*, where *y* is a value between 0 and 1 and *G* is the maximum gradient. The self diffusion coefficient of ethylene glycol is found by first calculating the temperature of the sample according to the empirical formula (2) from reference [5]:

$$T(\mathrm{K}) = 466.5 - 102.00(\Delta\delta) \quad (2)$$

where Δδ is the difference in chemical shift given in ppm between the two ^1H resonances of ethylene glycol. The spectrum of ethylene glycol with linewidths shimmed to the extent possible for this probe is shown in Supplementary Figure 9. The temperature obtained by measuring Δδ may then be related to the diffusion coefficient of ethylene glycol by a second empirical relation (3) from reference [6]:

$$D_{Et.Gly.} = exp(9.776 - \frac{3637}{T(\mathrm{K})}) \quad (3)$$

Maximum gradient strengths up to 6.18 T/m were measured by this method. By comparing the maximum gradient strength to the current (in amperes) applied to the gradient coil, the gradient efficiency was calculated to be 1.24 $Tm^{-1}A^{-1}$. The maximum gradient calculated at the current used for the PFG NMR experiments was measured to be 4.7 Tm^{-1} (Supplementary Figure 8).

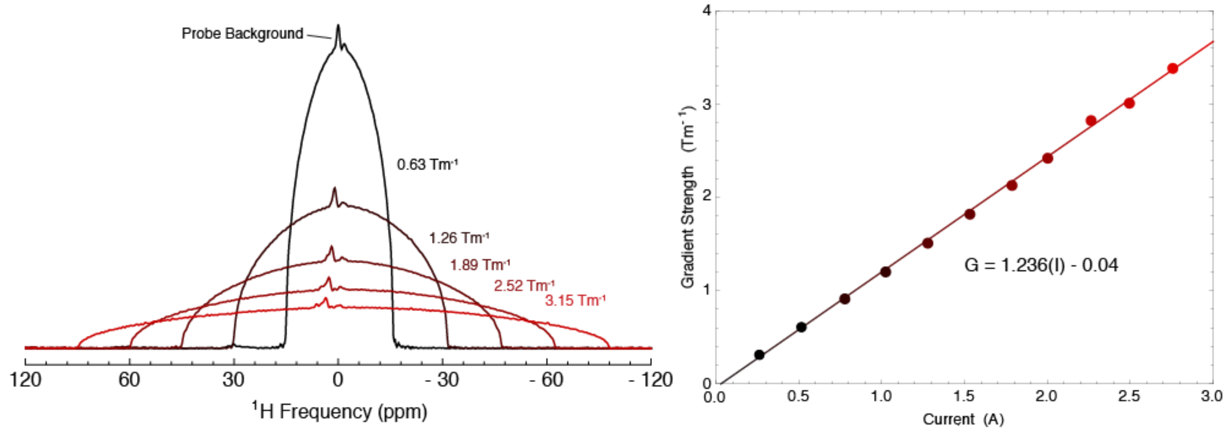

Supplementary Figure 10. 1D image of a 2.38 mm diameter cylindrical phantom of deionized water as a function of increasing current applied to the gradient coil, and plot of gradient strength as a function of increasing current with corresponding linear fit.

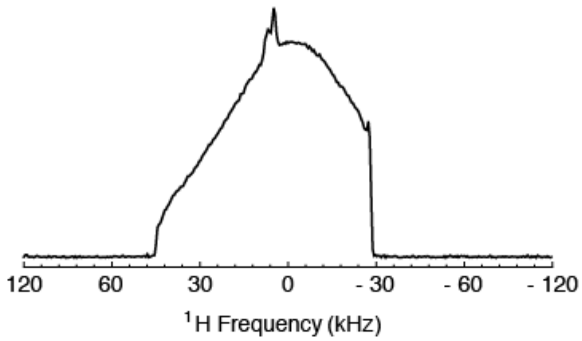

Supplementary Figure 11. 1D image of a 2.38 mm diameter cylindrical phantom of deionized water using an improperly wound gradient coil. The asymmetry of the image reveals the inhomogeneity of the gradient across the sample.

To corroborate the calibration of the gradient by this method, a phantom composed of a cylindrical sample of deionized water between two Teflon plugs within a 2.38 mm inner diameter glass tube and 3.75 mm in length was imaged using a spin echo 1D imaging sequence.[9] The resulting image from this measurement shows the distribution of water perpendicular to the long axis of the cylinder phantom, as well as resonances attributable to epoxy and polymer material ^{1}H background from the probe outside of the RF coil. The strength of the gradient may be measured by recording the frequency span of the image in Hz and then calculated according to equation (4):

$$\Delta f = \gamma g r \tag{4}$$

where $\gamma(^{1}H)$ = 42.577 MHz T^{-1}. Supplementary Figure 10 shows the 1D image as a function of increasing current applied. This method was viable for calculating the gradient efficiency up to about 4 Tm^{-1}, after which the signal-to-noise ratio was too low to accurately record the image. The gradient efficiency calculated via this method was found to be in agreement with the ethylene glycol method, yielding an efficiency of 1.24 $Tm^{-1}A^{-1}$. An additional benefit of imaging a phantom to calibrate a gradient for diffusion experiments is that ability to discern the homogeneity of the gradient over the sample space. The images in Supplementary Figure 10 are symmetric and depict the profile expected for a 1D projection of a cylinder imaged along its width. Early build attempts yielded gradient coils which resulted in asymmetric images of the phantom, due to improper winding (Supplementary Figure 11). 1D imaging provides a fast verification of a symmetrically wound gradient coil of this type.

NMR Experimental Parameters. ^{1}H PFG-STE NMR experiments at room temperature (23˚C as measured by ethylene glycol) were performed at 299.8 MHz using a 3.6 μs ^{1}H 90° pulse, a 1 ms sine-shaped gradient pulse width (δ) with 20 amplitude increments of the gradient pulses between 5 and 100% of 4.7 Tm^{-1}, and a 50 ms diffusion interval (Δ). 64 scans per increment were acquired, with an inter-scan relaxation delay of 6 s used for all experiments. Increments with signal area below 0.1% of the first increment were generally not included in fits to the data due to poor signal to noise.

Section 4: PFG NMR Signal Attenuation Plots and Tables of Self-Diffusion Coefficients

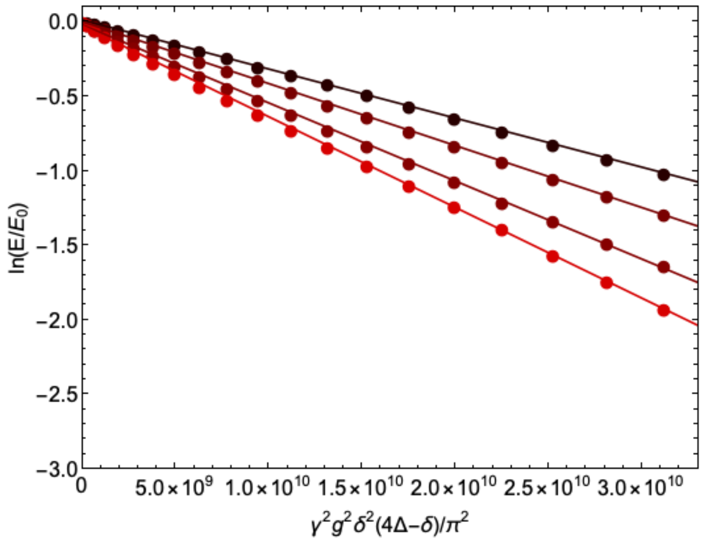

Supplementary Figure 12. Diffusion attenuation curves for DMF in MTV-MOFs with 5.1-A, 44.6, 59.2, and 84.0% NH_2-BDC. The change from red to black represents the increasing amount of NH_2-BDC linker in the structure.

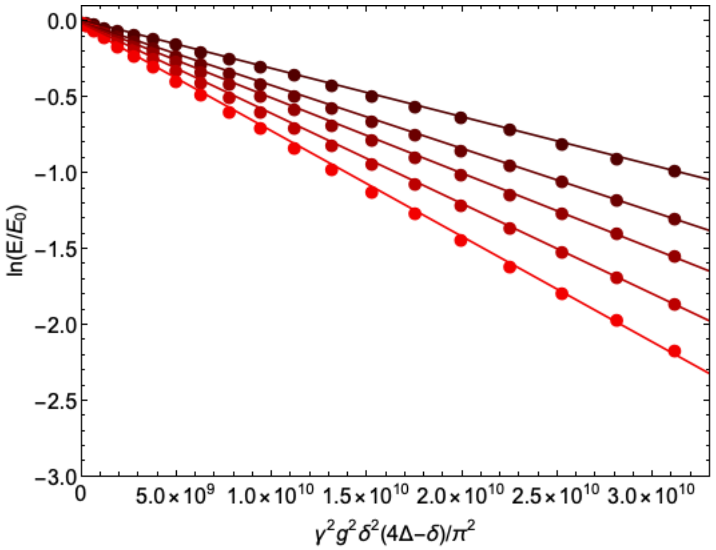

Supplementary Figure 13. Diffusion attenuation curves for DMF in MTV-MOFs with 1.4, 15.7-A, 36.4, 67, and 72.5% NH_2-BDC. The change from red to black represents the increasing amount of NH_2-BDC linker in the structure.

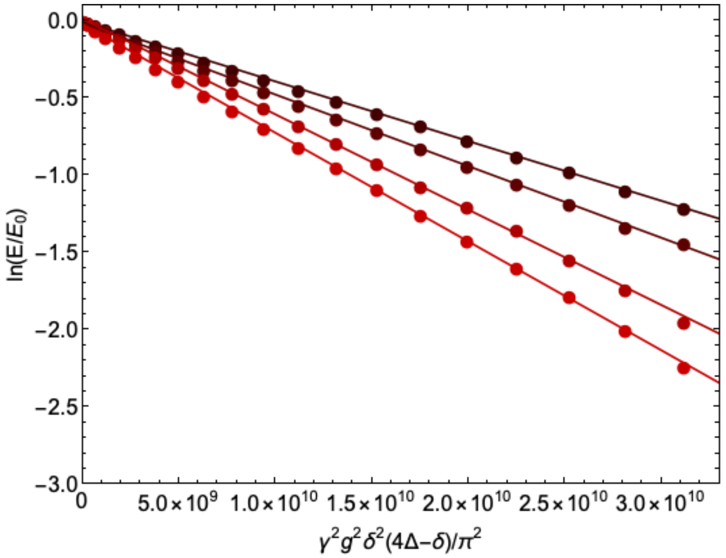

Supplementary Figure 14. Diffusion attenuation curves for DMF in MTV-MOFs with 10.7, 15.7-B, 70.6, and 75.1% NH_2-BDC. The change from red to black represents the increasing amount of NH_2-BDC linker in the structure.

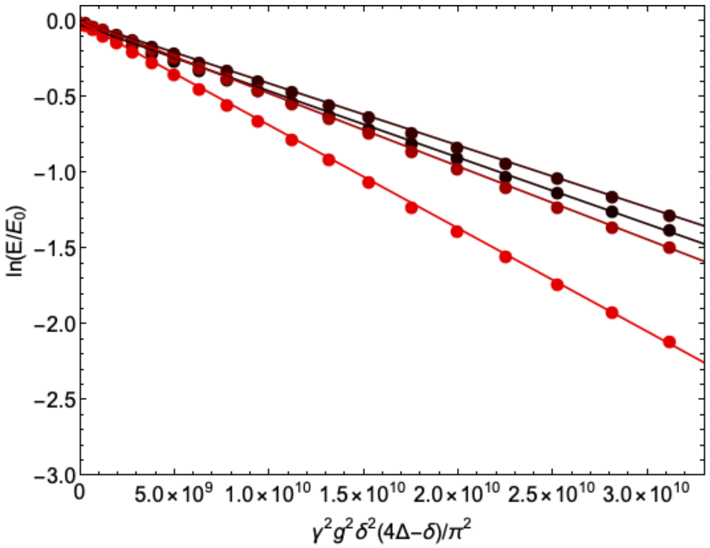

Supplementary Figure 15. Diffusion attenuation curves for DMF in MTV-MOFs with 5.1-B, 27.2, 79.2, and 85.0% NH_2-BDC. The change from red to black represents the increasing amount of NH_2-BDC linker in the structure.

Supplementary Table 1. Self-diffusion coefficients with standard error for DMF within the pores of the MTV-MOF samples. Values were obtained from fits of the data shown in Supplementary Figures 12-15, which correspond to points shown in Main Text Figure 2.

MTV-MOF Mole Fraction (% NH_2-BDC)	Self-Diffusion Coefficient of DMF (10^{-11} m^2s^{-1})
1.4	6.96 ± 0.02
5.1-A	6.82 ± 0.05
5.1-B	6.08 ± 0.02
10.7	7.04 ± 0.04
15.7-A	5.94 ± 0.03
15.7-B	6.17 ± 0.03
27.2	4.82 ± 0.01
36.4	4.96 ± 0.02
44.6	5.24 ± 0.03
59.2	4.16 ± 0.01
67	4.16 ± 0.02
70.6	4.64 ± 0.02
72.5	3.19 ± 0.01
75.1	3.87 ± 0.01
79.2	4.09 ± 0.01
84	3.29 ± 0.01
85	4.39 ± 0.04

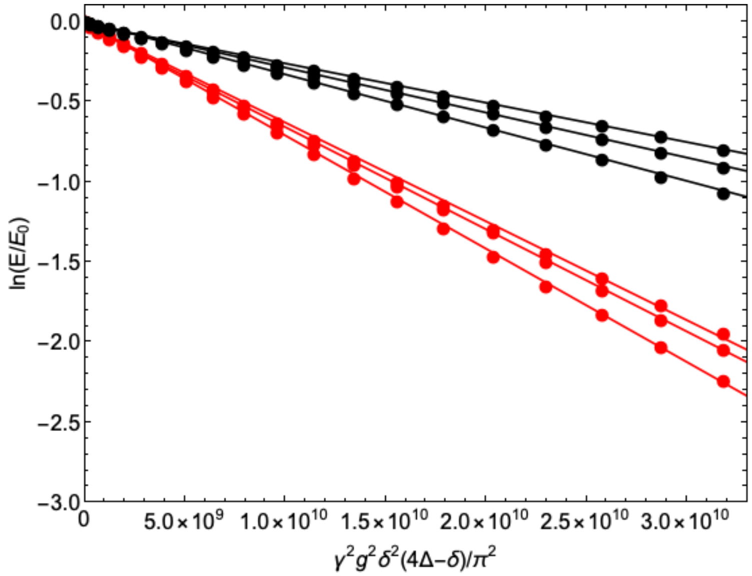

Supplementary Figure 16. Diffusion attenuation curves for DMF in three batches of MOF-5 (red) and three batches of IRMOF-3 (black).

Supplementary Table 2. Self-diffusion coefficients with standard error for DMF within the pores of three separate batches of MOF-5 and three separate batches of IRMOF-3 samples. Values were obtained from fits of the data shown in Supplementary Figure 16, which correspond to points shown in Main Text Figure 2.

Sample with Batch Label	Self-Diffusion Coefficient of DMF (10^{-11} m^2s^{-1})
MOF-5-A	7.06 ± 0.02
MOF-5-B	6.37 ± 0.02
MOF-5-C	6.15 ± 0.04
MOF-5 Average with Standard Deviation	6.53 ± 0.47
IRMOF-3-A	2.81 ± 0.01
IRMOF-3-B	3.32 ± 0.01
IRMOF-3-C	2.45 ± 0.02
IRMOF-3 Average with Standard Deviation	2.86 ± 0.44

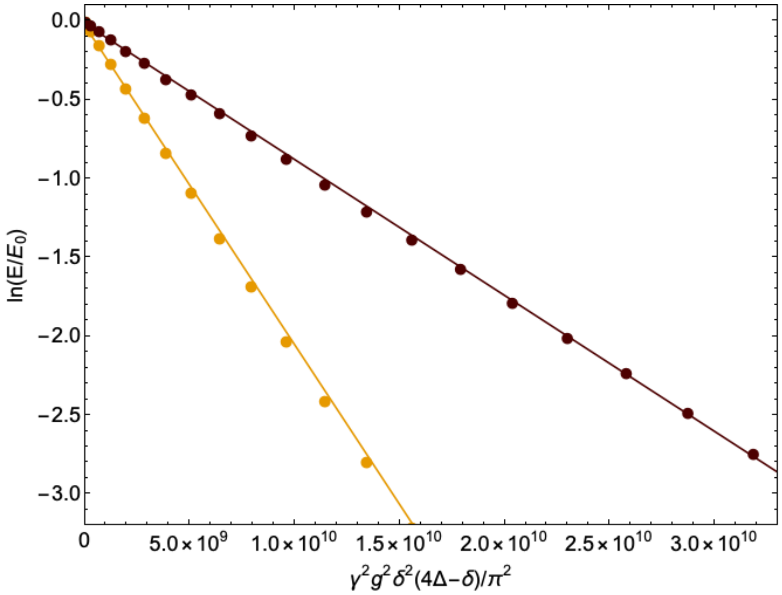

Supplementary Figure 17. Diffusion attenuation curves for benzene in MOF-5-B (yellow) and IRMOF-3-B (dark brown).

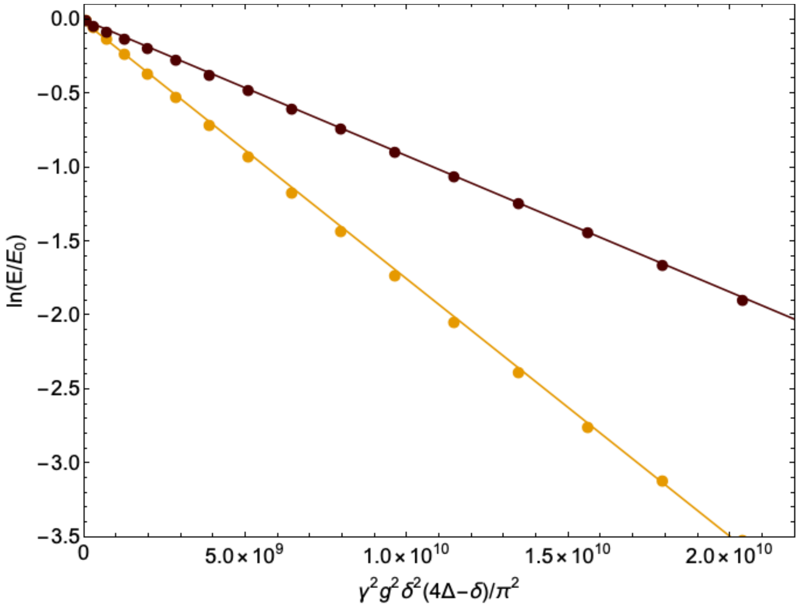

Supplementary Figure 18. Diffusion attenuation curves for toluene in MOF-5-B (yellow) and IRMOF-3-B (dark brown).

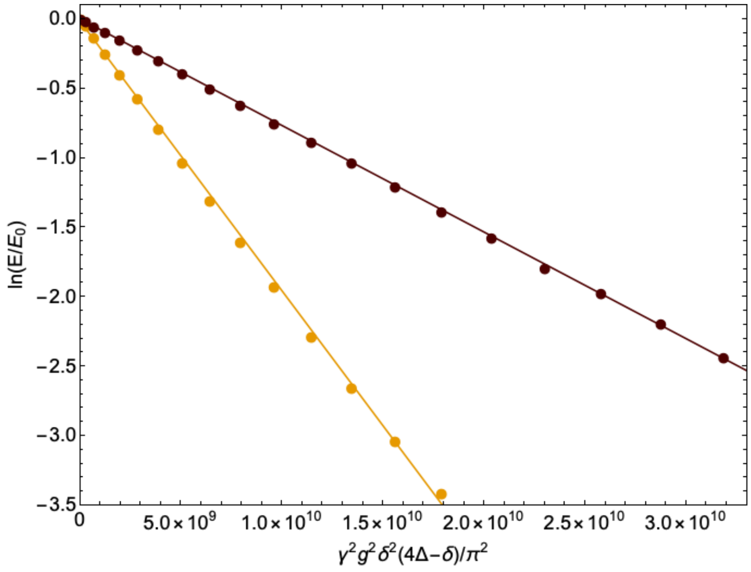

Supplementary Figure 19. Diffusion attenuation curves for p-xylene in MOF-5-B (yellow) and IRMOF-3-B (dark brown).

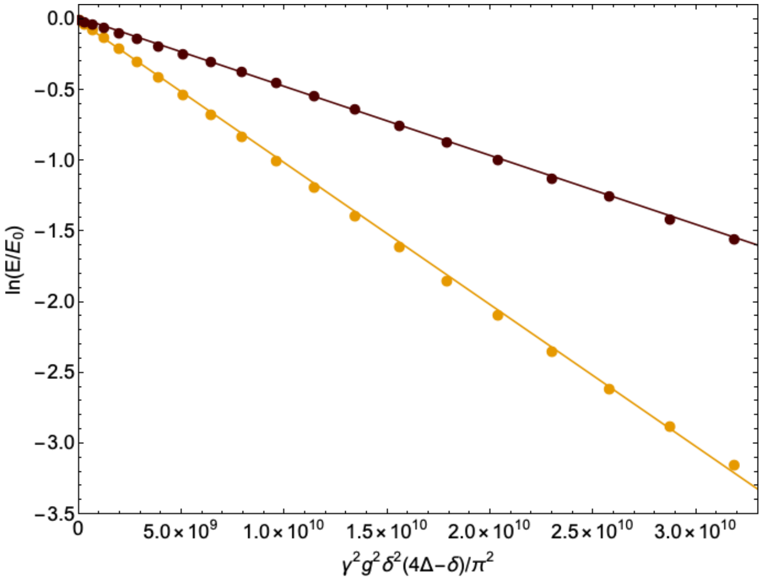

Supplementary Figure 20. Diffusion attenuation curves for m-xylene in MOF-5-B (yellow) and IRMOF-3-B (dark brown).

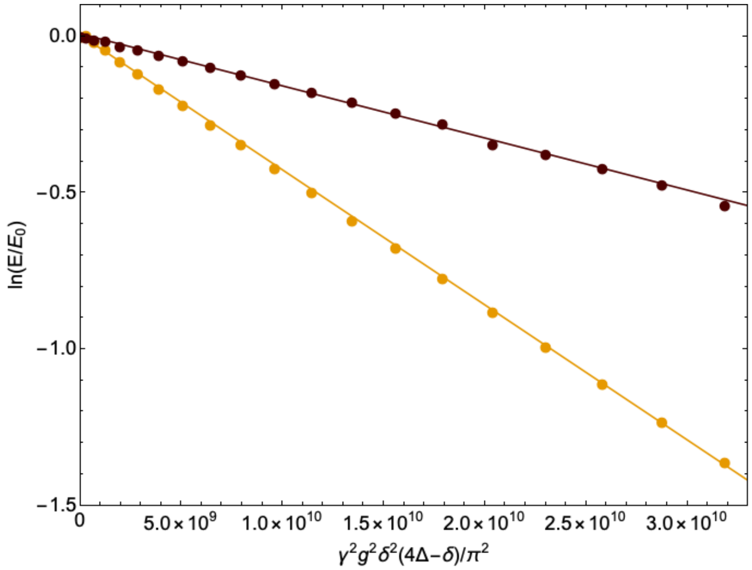

Supplementary Figure 21. Diffusion attenuation curves for o-xylene in MOF-5-B (yellow) and IRMOF-3-B (dark brown).

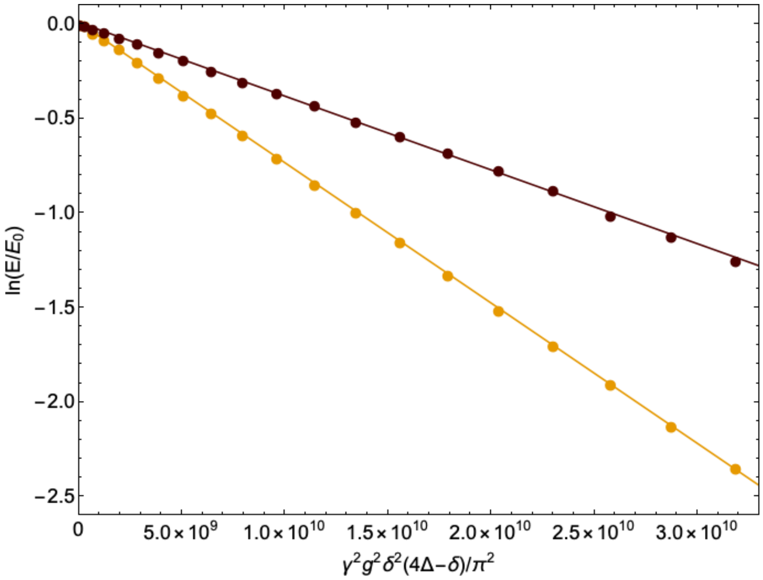

Supplementary Figure 22. Diffusion attenuation curves for anisole in MOF-5-B (yellow) and IRMOF-3-B (dark brown).

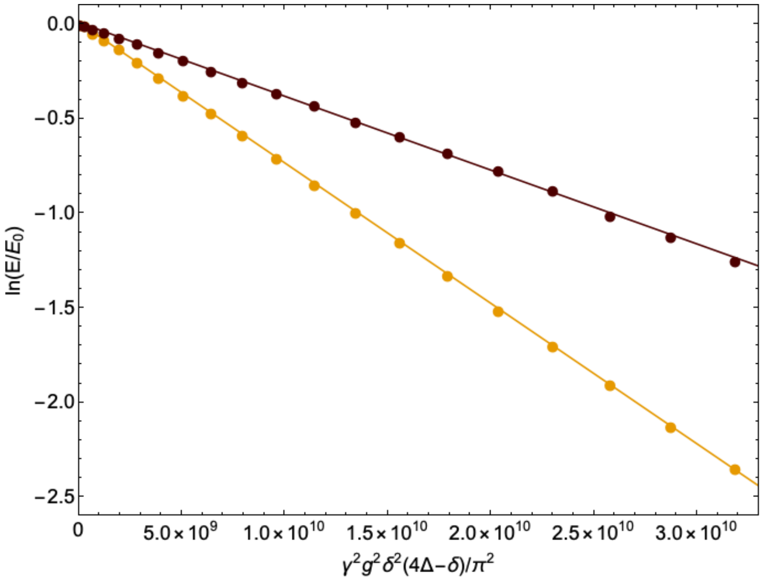

Supplementary Figure 23. Diffusion attenuation curves for 1,2,4-trimethylbenzene in MOF-5-B (yellow) and IRMOF-3-B (dark brown).

Supplementary Table 3. Self-diffusion coefficients with standard error for a series of solvents within the pores of MOF-5 (2) and IRMOF-3 (2). Values were obtained from fits of the data shown in Supplementary Figures 17-23. These values correspond to points shown in Main Text Figure 3.

Solvent	Self-Diffusion Coefficient of Neat Solvent (10^{-9} m^2s^{-1})	Self-Diffusion Coefficient within MOF-5 (10^{-11} m^2s^{-1})	Self-Diffusion Coefficient within IRMOF-3 (10^{-11} m^2s^{-1})	$D_{MOF-5}/D_{IRMOF-3}$
Benzene	2.04 ± 0.01	20.3 ± 0.2	8.62 ± 0.04	2.35 ± 0.03
Toluene	2.01 ± 0.02	17.4 ± 0.1	9.20 ± 0.02	1.89 ± 0.01
p-Xylene	1.94 ± 0.02	19.4 ± 0.2	7.67 ± 0.02	2.52 ± 0.03
m-Xylene	1.78 ± 0.01	10.0 ± 0.1	4.88 ± 0.02	2.05 ± 0.02
o-Xylene	1.44 ± 0.03	4.32 ± 0.02	1.67 ± 0.02	2.59 ± 0.03
DMF	1.51 ± 0.03	6.37 ± 0.02	3.32 ± 0.01	1.92 ± 0.01
Anisole	1.12 ± 0.01	7.42 ± 0.01	3.90 ± 0.02	1.90 ± 0.01
1,2,4-trimethylbenzene	1.33 ± 0.01	3.52 ± 0.01	1.30 ± 0.01	2.71 ± 0.02

Section 5: References

[1] P.T. Callaghan, M.E. Komlosh, M. Nyden, *Journal of Magnetic Resonance*, **1998**, *133*, 177-182.

[2] A.C. Wright, H. Bataille, H.H. Ong, S.L. Wehrli, H.K. Song, F.W. Wehrli, *Journal of Magnetic Resonance*, **2007**,*186*, 17–25.

[3] W.S. Price, K. Hayamizu, H. Ide, Y. Arata, *Journal of Magnetic Resonance*, **1999**, *139*, 205–212.

[4] A.L. Van Geet, *Analytical Chemistry*, **1968**, *40*, 2227–2229.

[5] C. Amman, P. Meier, A.E. Merbach, *Journal of Magnetic Resonance,* **1982**; *46*, 319–321.

[6]. W.M. Spees, S.K. Song, J.R. Garbow, J.J. Neil, J.J. Ackerman, *Magnetic Resonance in Medicine*, **2012**, *68*, 319-324.

[7] W.S. Price, *Concepts in Magnetic Resonance: An Educational Journal*, **1998**, *10*, 197-237.

[8] E.O. Stejskal, J.E. Tanner, *The Journal of Chemical Physics*, **1965***, 42*, 288-292.

[9] R.W. Brown, E.M. Haacke, Y.C.N. Cheng, M.R. Thompson, R. Venkatesan, **2014**. *Magnetic resonance imaging: physical principles and sequence design*. Section 9.4.2, John Wiley & Sons.
